# Heat remains unaccounted for in thermal physiology and climate change research

**DOI:** 10.12688/f1000research.10554.2

**Published:** 2017-03-15

**Authors:** Andreas D. Flouris, Glen P. Kenny

**Affiliations:** 1 FAME Laboratory, Department of Exercise Science, University of Thessaly, Trikala, Greece; 2 Human and Environmental Physiology Research Unit, School of Human Kinetics, University of Ottawa, Ottawa, ON, Canada

**Keywords:** global warming, hiatus, temperature, ocean heat uptake, hyperthermia

## Abstract

In the aftermath of the Paris Agreement, there is a crucial need for scientists in both thermal physiology and climate change research to develop the integrated approaches necessary to evaluate the health, economic, technological, social, and cultural impacts of 1.5°C warming. Our aim was to explore the fidelity of remote temperature measurements for quantitatively identifying the continuous redistribution of heat within both the Earth and the human body. Not accounting for the regional distribution of warming and heat storage patterns can undermine the results of thermal physiology and climate change research. These concepts are discussed herein using two parallel examples: the so-called slowdown of the Earth’s surface temperature warming in the period 1998-2013; and the controversial results in thermal physiology, arising from relying heavily on core temperature measurements. In total, the concept of heat is of major importance for the integrity of systems, such as the Earth and human body. At present, our understanding about the interplay of key factors modulating the heat distribution on the surface of the Earth and in the human body remains incomplete. Identifying and accounting for the interconnections among these factors will be instrumental in improving the accuracy of both climate models and health guidelines.

## Introduction

The Agreement reached in Paris during December 2015, under the auspices of the United Nations Framework Convention on Climate Change, binds countries to “…pursue efforts to limit the [global] temperature increase to 1.5°C above pre-industrial levels”. The same document also invites the Intergovernmental Panel on Climate Change (IPCC) to “…provide a special report in 2018 on the impacts of global warming of 1.5°C above pre-industrial levels and related global greenhouse gas emission pathways” (full document: Paris Agreement, FCCC/CP/2015/10/Add.1, annex. UNFCCC secretariat. Retrieved 20 January 2017). In doing so, the IPCC must provide useful and robust evidence, which can be a challenge, as previously suggested ( Hulme, 2016), given the limited analyses conducted thus far on the global and regional impacts of 1.5°C warming. Another challenge for the IPCC, and the scientific community, is to develop the integrated approaches necessary to evaluate the health, economic, technological, social, and cultural impacts of 1.5°C warming. As illustrated by a recent scientific conundrum (described in the following section), the concept of heat should be central to these integrated approaches. In this light, the objective of this article is to explore the fidelity of remote temperature measurements for quantitatively identifying the continuous redistribution of heat within both the Earth and human body.

## Unaccounted heat in climate research

Hailed as “one of the biggest mysteries in climate science” ( Tollefson, 2013), the slowdown of the Earth’s surface temperature warming in the period 1998–2013 undermined, for a number of years, the idea that human-generated greenhouse gases are the main driver and cause of climate change ( Nieves *et al.*, 2015). During this period, the existing climate models were unable (on average) to reproduce the observed atmospheric temperature trend. Several theories were proposed to explain this surface temperature “hiatus”. One of the first explanations put forth was that it is normal for warming rates to plateau occasionally ( Trenberth, 2015). Other factors that were extensively investigated included the increased volcanic activity and the high levels of air pollution in China during the hiatus period, since atmospheric particles reflect more of the Sun’s energy back into space ( Solomon *et al.*, 2011). The ever-increasing decline in solar activity since 2000 was also considered as a possibility, since it reduces the amount of energy reaching the Earth ( Ineson *et al.*, 2015). Ultimately, in 2015, Nieves and colleagues showed that what was interpreted as a change in the planet’s warming rate was, in fact, a redistribution of heat within the ocean ( Nieves *et al.*, 2015). Specifically, Nieves *et al.*, showed that heat moved from the surface of the Pacific Ocean to the deeper layers of the Western Pacific and Indian Oceans, a finding that was confirmed using a wealth of observational and simulated data. As a result, the net ocean heat uptake remains dangerously high ( Nieves *et al.*, 2015), and the rate of global warming may have actually increased during the hiatus period ( IPCC, 2014). The observed record-breaking temperatures throughout 2015 and 2016 ( https://www.nasa.gov/press-release/nasa-noaa-data-show-2016-warmest-year-on-record-globally) support the findings of Nieves *et al*.

Research aiming to understand the 1998–2013 hiatus significantly benefited from studies investigating the causes of a much longer hiatus observed from the 1950s to the 1970s. During this period, global mean surface temperature remained approximately constant despite increased burden from anthropogenic factors. Interestingly, as in the case of the 1998–2013 hiatus, the ocean heat uptake was a very important contributing factor to this longer offsetting of the warming rate ( England *et al.*, 2014). This is because ocean circulation changes have a vast impact on the geographical distribution of warming and heat storage patterns, which ultimately affect the planet’s surface climate ( Nieves *et al.*, 2015). Measuring atmospheric temperature at specific regions can only indicate regional changes in heat content and – as in the case of the 1998–2013 hiatus – can lead to wrong conclusions about the planet’s warming rate.

## Unaccounted heat in thermal physiology research

Although the Earth is a “passive system” with no active regulation of temperature or heat content (based on current knowledge, that is), the effect of ocean heat uptake on the geographical distribution of warming and the heat storage patters on the surface of the planet closely mirror thermodynamic processes within the human body. Blood within the circulatory system is constantly redistributed, not only to meet the demands of metabolic and immune processes, but also to move heat from/to specific regions and maintain thermal homeostasis ( Flouris *et al.*, 2006; Flouris & Cheung, 2009; Kenny & Jay, 2013; Kiyatkin, 2010). These blood flow adaptations can reach dramatic proportions in the human body. For instance, during rest in thermoneutral conditions, 0.5 L/min of blood (5–10% of cardiac output) supplys the skin ( Lossius *et al.*, 1993). Nevertheless, during heat stress, up to 8 L/min of blood (50–70% of cardiac output) is directed to the cutaneous circulation, mainly by restricting visceral and renal blood flow ( Lossius *et al.*, 1993). These enormous changes in blood flow have a vast effect on the regional distribution of heat and the heat storage patterns, which ultimately affect the entire body’s thermal homeostasis. Therefore, measuring temperature in specific regions of the body, such as the rectum, esophagus, or the visceral organs, can only indicate regional changes in heat content ( Flouris & Cheung, 2010; Flouris & Cheung, 2011; Kenny *et al.*, 2015; Kenny *et al.*, 2017; Taylor *et al.*, 2014; Webb, 1986). Moreover, peripheral tissues can tolerate greater changes in heat with less threat to the health and survivability of the organism, whereas increases in specific organ structures such as the brain can threaten organism health ( Kiyatkin, 2010). Nevertheless, for more than 100 years, scientists have been using single region temperature measurements to make assumptions about the thermal strain across the entire body. Moreover, the currently recommended criterion when addressing heat-related health risks is a single temperature measurement in the rectum ( World Meteorological Organization and World Health Organization, 2015).

A recent paper on occupational heat exposure ( Meade *et al.*, 2016) provides an example of the limitations inherent in single temperature measurements used to assess whole-body thermal strain. In hot environments, industries must take preventive measures to protect their workers against heat-related injury and illness. To determine what protections should be used, they may follow guidelines from bodies of knowledgeable experts. One such set of guidelines comes from the American Conference of Governmental Industrial Hygienists ( ACGIH, 2007); they recommend that industries employ the Threshold Limit Values (TLV) for work in hot environments. The TLV consider both environmental conditions and work demands, with the goal of maintaining the internal body temperatures of workers within safe limits. However, it is unclear if these guidelines adequately protect workers. While the history of the TLV guidelines starts in 1971 ( ACGIH, 2007), to the best of our knowledge, the aforementioned recent paper is the only one that directly assessed the influence of the work exposure limits outlined in the TLV on core temperature responses during work and the associated changes in whole-body heat content. This study applied the TLV recommendations for work-rest periods by having a group of participants perform moderate intensity work bouts in progressively hotter environments. According to the TLV, as environmental heat levels increased, the recovery periods between work bouts were lengthened ( Meade *et al.*, 2016). Once these guidelines are applied, the TLV predict that the participant’s core temperature should be minimally affected. Yet, the core temperatures of the young physically active adults tested in this study rose continuously. Overall, the findings demonstrated that, under the work conditions tested, the TLV do not adequately protect workers from potentially dangerous increases in their internal temperatures. As mentioned above, these findings are likely ascribable to a heterogeneous distribution of heat within the body’s ‘core’ tissues (i.e., organs, muscles) ( Taylor *et al.*, 2014), which may be influenced by the profound thermoregulatory and cardiovascular (the majority of heat transfer within the body occurs through convective transfer via blood) alterations associated with recovery from exercise ( Halliwill *et al.*, 2014; Kenny & Journeay, 2010; Kenny & Jay, 2013). If this peripheral heat storage is transferred from the tissues to the body’s core, it presents an increased risk of heat related illness and injury during work. Therefore, the TLV guidelines should be revised, especially given the warming climate and the increase in the frequency and intensity of extreme heat events.

## Heat parallels in the two disciplines

Heat distribution (and its associated temperature variation) is important in both the Earth and the human body and our understanding and measurements of it need to be improved in the interest of human health (since human thermal homeostasis is largely dependent on environmental temperature). In this light, recognition that ocean heat uptake plays a key role in modulating human-caused global surface warming has been one of the many valuable ancillary benefits of research aiming to understand the 1998–2013 hiatus. Identifying and accounting for errors in ocean heat uptake estimations has been vital in this improved understanding ( Fyfe *et al.*, 2016) and will be instrumental in improving the accuracy of future climate predictions. For instance, climate models accounting for these recent advances suggest a transition to a positive phase of the Interdecadal Pacific Oscillation, which will increase the warming rate of global surface temperature ( Hawkins *et al.*, 2014; Thoma *et al.*, 2015). Similarly to climate studies, thermal physiology research employing calorimetric methods has shown that temperature measurements at a single region of the body’s core do not (on average) reflect whole-body thermal strain [( Flouris & Cheung, 2010; Kenny *et al.*, 2013; Kenny *et al.*, 2017; Meade *et al.*, 2016; Stapleton *et al.*, 2014; Stapleton *et al.*, 2015; Webb, 1986), including reviews ( Benzinger *et al.*, 1961; Taylor *et al.*, 2014; Webb, 1995)]. In addition to their limited accuracy, such measurements contain a significant time lag, which makes them less effective for scientific purposes and potentially problematic when used in health-related settings. Indeed, core temperature readings are notorious for responding with delays of 10–30 minutes ( Kenny & Jay, 2013), even when the human body undergoes extreme discomfort, such as a sudden passive immersion in 12°C water ( Flouris & Cheung, 2009). Interestingly, a similar time lag exists in the Earth’s warming rate phenomenon, since 80% of the heat added to the climate system is being taken up by the ocean ( IPCC, 2014). As a result, even if greenhouse gas concentrations were to drastically reduce, the planet would continue to warm for many decades ( IPCC, 2014).

## Concluding remarks

Scientists in thermal physiology and climate change research are measuring temperature but, very often, they are missing the heat. Relying on indicators that provide a less accurate and/or delayed view of a natural phenomenon is dangerous. In the case of climate change research, interpreting the slowdown of atmospheric temperature rise during 1998–2013 as a change in the planet’s warming rate would have given way to lax policies in environmental monitoring. In the 15 years required to identify the key factors involved, obtain more accurate data, and reassess the situation, these policies could have produced devastating effects and pushed the planet’s climate into unchartered territory. In a similar way, interpreting the minimal temperature changes often observed in a single region of the body’s core as a lack of whole-body thermal strain, gives way to an impassable abyss of controversial results in scientific experiments and, even worse, ineffective guidelines for work in hot environments, as well as delayed application of treatment in occupational or clinical settings.

The concept of heat is of major importance for the integrity of systems, such as the Earth ( IPCC, 2014; Nieves *et al.*, 2015) and the human body ( Borden & Cutter, 2008; Flouris & Piantoni, 2015; Luber & McGeehin, 2008). At present, our understanding about the interplay of key factors modulating the heat distribution on the surface of the Earth and in the human body remains incomplete. Identifying and accounting for the interconnections among these factors will be instrumental in improving the accuracy of both climate models and health guidelines. In the aftermath of the Paris Agreement, there is a need for scientists in both thermal physiology and climate change research to embrace integrated approaches that provide comprehensive views of the natural phenomena under study, taking into account the distribution of warming and heat storage patterns. For on that may depend the future in these scientific disciplines and, possibly, the future of the planet.
