# Supplementary material for: Heat remains unaccounted for in thermal physiology and climate change research
Source: F1000Res. 2017 Mar 15;6:221. Originally published 2017 Mar 6. [Version 2] doi: 10.12688/f1000research.10554.2 (PMC5357026; doi:10.12688/f1000research.10554.2)
Supplement: Peer review history

# Peer review: Referee response for version 2

**DOI:** 10.5256/f1000research.12019.r21009

**Published:** 2017-03-16

**Authors:** Eugene A. Kiyatkin

**Affiliations:** 1 Behavioral Neuroscience Branch, National Institute on Drug Abuse – Intramural Research Program, National Institutes of Health (NIH), Baltimore, MD, USA

The Authors properly responded to all comments of our previous review.

I have read this submission. I believe that I have an appropriate level of expertise to confirm that it is of an acceptable scientific standard.

# Peer review: Referee response for version 1

**DOI:** 10.5256/f1000research.11373.r20720

**Published:** 2017-03-08

**Authors:** Eugene A. Kiyatkin, Keaton T. Cameron-Burr

**Affiliations:** 1 Behavioral Neuroscience Branch, National Institute on Drug Abuse – Intramural Research Program, National Institutes of Health (NIH), Baltimore, MD, USA; 2 National Institute of Drug Abuse, Intramural Research Program, National Institutes of Health (NIH), Baltimore, MD, USA

**Title:** Heat remains unaccounted for in thermal physiology and climate change research

 Comments:

  

- This opinion piece touches on an important issue. It is critical to accurately measure the redistribution of heat within both climate and physiological systems. The authors correctly note the interconnectedness of the two: as the earth warms, heat-related health risks will rise as the dynamics of energy transfer between organism and environment change. Standards to protect workers and the environment should be updated to reflect the most accurate and helpful measurements and information as is possible.

 

 Major comments:

  

- The authors draw a parallel between heat distribution in human and heat distribution within climate systems throughout the piece. While I see the analogy, it might be best to reign in this comparison in some places, specifically, on page 3 under the heading “Heat parallels in the two disciplines”. While the point is well taken, the human body has many mechanisms that actively work to maintain thermo-homeostasis. The earth, on the other hand, is a passive system with no “goal temperature”, as it were. Would it be better simply to state that temperature distribution is important in both systems and that our understanding and measurements of it need to be improved in the interest of human health? Given that heat distribution in humans is dependent on the temperature of the environment, there is a strong argument to be made linking these two variables without drawing a comparison between an actively regulated homeostatic system and system in which heat distribution occurs through passive mechanisms. The first paragraph of “Unaccounted heat in thermal physiology research” contains this analogy as well.

 

 Minor:

  

- The authors put forward that the findings of Nieves *et al*. were confirmed by increased temperatures in 2015 and 2016 (Page 2). When considering global trends, single year data is a fraught method of proof given the degree of between-year variability in temperature. By the same logic, the climate change slowdown from 1998-2013 discussed in this paper was used to justify a lack of action around climate change.
- Some slightly awkward phrasing persists in this article. For example, the first sentence of the second paragraph on page 2 under “Unaccounted heat in climate research” could be re-written as “Research aiming to understand the 1998-2013 hiatus significantly benefited from studies investigating the causes…”. The sentence, “This is not surprising.” Under the same heading on page 2, does not add to the paragraph and could be cut.
- “Unaccounted heat in thermal physiology research” could benefit from some discussion of the importance of brain temperature (Kiyaktin 2010 for review ^1^). Individuals may experience robust changes in brain temperature during behavioral activation such as manual labor or intense exercise that can result, under conditions of diminished heat dissipation, in pathological hyperthermia ^2^. Hyperthermia increases permeability of the blood-brain barrier, predisposing individuals to the development of vasogenic edema and damage of brain cells. Increased global temperature may also pose increased health risks for people using psychomotor stimulant drugs of abuse, the effects of which are greatly potentiated by increased ambient temperature. This is critical when considering issues of heat distribution in which peripheral tissues can tolerate greater changes in heat with less threat to the health and survivability of the organism, whereas increases in specific organ structures such as the brain can threaten organism health.

We have read this submission. We believe that we have an appropriate level of expertise to confirm that it is of an acceptable scientific standard, however we have significant reservations, as outlined above.

## References

[1] KiyatkinEA: Brain temperature homeostasis: physiological fluctuations and pathological shifts. *Front Biosci (Landmark Ed)*.2010;15:73-92 2003680810.2741/3608PMC3149793

[2] KiyatkinEASharmaHS: Environmental conditions modulate neurotoxic effects of psychomotor stimulant drugs of abuse. *Int Rev Neurobiol*.2012;102: 10.1016/B978-0-12-386986-9.00006-5 147-71 10.1016/B978-0-12-386986-9.00006-5 22748829PMC3687356

# Response

**Published:** 2017-03-09

**Authors:** Andreas Flouris

**Affiliations:** FAME Laboratory, Department of Exercise Science, University of Thessaly, Greece

We wish to thank you for reviewing the manuscript and for your constructive and helpful comments. We made appropriate changes in the paper based on your comments. The appropriate responses to all points that you raised appear below with each of your comments in *underlined italics* and our responses in **bold** **font**. **Bold underlined font** is used to indicate revised parts of the text.

*Comments:*

*This opinion piece touches on an important issue. It is critical to accurately measure the redistribution of heat within both climate and physiological systems. The authors correctly note the interconnectedness of the two: as the earth warms, heat-related health risks will rise as the dynamics of energy transfer between organism and environment change. Standards to protect workers and the environment should be updated to reflect the most accurate and helpful measurements and information as is possible.*

**Thank you for your encouraging comments.**

*Major comments:*

*The authors draw a parallel between heat distribution in human and heat distribution within climate systems throughout the piece. While I see the analogy, it might be best to reign in this comparison in some places, specifically, on page 3 under the heading “Heat parallels in the two disciplines”. While the point is well taken, the human body has many mechanisms that actively work to maintain thermo-homeostasis. The earth, on the other hand, is a passive system with no “goal temperature”, as it were. Would it be better simply to state that temperature distribution is important in both systems and that our understanding and measurements of it need to be improved in the interest of human health? Given that heat distribution in humans is dependent on the temperature of the environment, there is a strong argument to be made linking these two variables without drawing a comparison between an actively regulated homeostatic system and system in which heat distribution occurs through passive mechanisms. The first paragraph of “Unaccounted heat in thermal physiology research” contains this analogy as well.*

**We agree with your observation. Specific comments were added in the relevant sections of the paper to improve clarity.**

**1 ^st^ paragraph of section “Unaccounted heat in thermal physiology research”:**

**“ Although the Earth is a “passive system” with no active regulation of temperature or heat content (based on current knowledge, that is),…”**

**1 ^st^ paragraph of section “Heat parallels in the two disciplines”:**

**“ Heat distribution (and its associated temperature variation) is important in both the Earth and the human body and our understanding and measurements of it need to be improved in the interest of human health (since human thermal homeostasis is largely dependent on environmental temperature).”**

*Minor:*

*The authors put forward that the findings of Nieves et al. were confirmed by increased temperatures in 2015 and 2016 (Page 2). When considering global trends, single year data is a fraught method of proof given the degree of between-year variability in temperature. By the same logic, the climate change slowdown from 1998-2013 discussed in this paper was used to justify a lack of action around climate change.*

**To improve clarity, we revised the 1 ^st^ paragraph of section “Unaccounted heat in climate research” as follows:**

**“Specifically, Nieves et al., showed that heat moved from the surface of the Pacific Ocean to the deeper layers of the Western Pacific and Indian Oceans, a finding that was confirmed using a wealth of observational and simulated data. As a result, the net ocean heat uptake remains dangerously high (Nieves et al., 2015), and the rate of global warming may have actually increased during the hiatus period (IPCC, 2014). The observed record-breaking temperatures throughout 2015 and 2016 (https://www.nasa.gov/press-release/nasa-noaa-data-show-2016-warmest-year-on-record-globally) support the findings of Nieves et al.”**

*Some slightly awkward phrasing persists in this article. For example, the first sentence of the second paragraph on page 2 under “Unaccounted heat in climate research” could be re-written as “Research aiming to understand the 1998-2013 hiatus significantly benefited from studies investigating the causes…”. The sentence, “This is not surprising.” Under the same heading on page 2, does not add to the paragraph and could be cut.*

**As suggested, the following revisions were done:**

**“Research aiming to understand the 1998–2013 hiatus significantly benefited from studies investigating the causes of a much longer hiatus observed from the 1950s to the 1970s.”**

**“This is because ocean circulation…”**

*“Unaccounted heat in thermal physiology research” could benefit from some discussion of the importance of brain temperature (Kiyaktin 2010 for review1). Individuals may experience robust changes in brain temperature during behavioral activation such as manual labor or intense exercise that can result, under conditions of diminished heat dissipation, in pathological hyperthermia2. Hyperthermia increases permeability of the blood-brain barrier, predisposing individuals to the development of vasogenic edema and damage of brain cells. Increased global temperature may also pose increased health risks for people using psychomotor stimulant drugs of abuse, the effects of which are greatly potentiated by increased ambient temperature. This is critical when considering issues of heat distribution in which peripheral tissues can tolerate greater changes in heat with less threat to the health and survivability of the organism, whereas increases in specific organ structures such as the brain can threaten organism health.*

**As suggested, we added the following sentence in the 1 ^st^ paragraph of the section entitled “Unaccounted heat in thermal physiology research”:**

**“ Moreover, peripheral tissues can tolerate greater changes in heat with less threat to the health and survivability of the organism, whereas increases in specific organ structures such as the brain can threaten organism health (Kiyatkin, 2010).”**

# Response

**Published:** 2017-03-16

**Authors:** Eugene A. Kiyatkin

**Affiliations:** NIDA-IRP, NIH, USA

The Authors properly responded to all comments raised in our review.

# Peer review: Referee response for version 1

**DOI:** 10.5256/f1000research.11373.r20753

**Published:** 2017-03-07

**Authors:** Juha Oksa

**Affiliations:** 1 Physical Work Capacity team, Finnish Institute of Occupational Health, Oulu, Finland

The opinion article by Dr’s Flouris and Kenny nicely describes the similarities in the changes of heat content pattern between the Earth and the human body. The difference between these two is naturally the time frame, the response of the Earth being several years and that of the human, minutes.

 Given that Earth is warming, extreme heat wave episodes are increasing and the study the authors are referring to (Meade *et al.* 2016 ^1^) points to the possibility that the existing TLV guidelines may not be sufficient in protecting labor force from excessive heat strain (as the authors state). Therefore, a re-evaluation of the current TLV guidelines might be desirable.

 However, it would also be desirable to see previous evaluations regarding the usability and effectiveness of the current guidelines. Has it been successful in preventing excessive heat strain? If not, what has been the causes? This might increase the justification for re-evaluation of the current TLV guidelines.

I have read this submission. I believe that I have an appropriate level of expertise to confirm that it is of an acceptable scientific standard.

## References

[1] MeadeRDPoirierMPFlourisADHardcastleSGKennyGP: Do the Threshold Limit Values for Work in Hot Conditions Adequately Protect Workers?. *Med Sci Sports Exerc*.2016;48(6) : 10.1249/MSS.0000000000000886 1187-96 10.1249/MSS.0000000000000886 26938043

# Response

**Published:** 2017-03-09

**Authors:** Andreas Flouris

**Affiliations:** FAME Laboratory, Department of Exercise Science, University of Thessaly, Greece

We wish to thank you for reviewing the manuscript and for your constructive and helpful comments. We made appropriate changes in the paper based on your comments. The appropriate responses to all points that you raised appear below with each of your comments in *underlined italics* and our responses in **bold** **font**. **Bold font** is used to indicate revised parts of the text.

*The opinion article by Dr’s Flouris and Kenny nicely describes the similarities in the changes of heat content pattern between the Earth and the human body. The difference between these two is naturally the time frame, the response of the Earth being several years and that of the human, minutes. Given that Earth is warming, extreme heat wave episodes are increasing and the study the authors are referring to (Meade et al. 20161) points to the possibility that the existing TLV guidelines may not be sufficient in protecting labor force from excessive heat strain (as the authors state). Therefore, a re-evaluation of the current TLV guidelines might be desirable.*

**Thank you for your encouraging comments.**

*However, it would also be desirable to see previous evaluations regarding the usability and effectiveness of the current guidelines. Has it been successful in preventing excessive heat strain? If not, what has been the causes? This might increase the justification for re-evaluation of the current TLV guidelines.*

**The history of the TLV guidelines starts in 1971 (ACGIH, 2007). However, to the best of our knowledge, the study referenced in our paper is the only one that directly assessed the influence of the work exposure limits outlined in the TLV on core temperature responses during work and the associated changes in whole-body heat content. To clarify this further, we added a relevant comment in the paper [2 ^nd^ paragraph of section “Unaccounted heat in thermal physiology research”]:**

**“While the history of the TLV guidelines starts in 1971 (ACGIH, 2007), to the best of our knowledge, the aforementioned recent study is the only one that directly assessed the influence of the work exposure limits outlined in the TLV on core temperature responses during work and the associated changes in whole-body heat content.”**
